# Large language model-assisted CAD-RADS classification: structured report extraction, accuracy, and clinical decision-making risk of ChatGPT-4o/5 and DeepSeek-R1

**DOI:** 10.3389/fcvm.2026.1909068

**Published:** 2026-10-09

**Authors:** Haorong Rong, Xiyi Huang, Xinjie Chen, Lizhu Ouyang, Yuelu Li, Xin Lin, Jialing Pan, Yunjing Li, Fan Xie, Fusheng Ouyang, Qiugen Hu, Baoliang Guo

**Affiliations:** 1 Department of Radiology, The Eighth Affiliated Hospital, Southern Medical University, Foshan, Guangdong, China; 2 Department of Clinical Laboratory, Lecong Hospital of Shunde, Foshan, Guangdong, China; 3 Department of Clinical Laboratory, The Eighth Affiliated Hospital, Southern Medical University, Foshan, Guangdong, China; 4 Department of Ultrasound, The Eighth Affiliated Hospital, Southern Medical University, Foshan, Guangdong, China

**Keywords:** CAD-RADS, ChatGPT, CT angiography, DeepSeek, large language models

## Abstract

**Objective:**

To compare ChatGPT-4o, ChatGPT-5, and DeepSeek-R1 in assigning main Coronary Artery Disease Reporting and Data System (CAD-RADS) categories from findings-only coronary computed tomography angiography (CCTA) reports and to characterize management-relevant classification errors.

**Methods:**

This retrospective diagnostic-accuracy study included 2,100 category-balanced findings-only CCTA reports (300 per CAD-RADS category). Two experienced CCTA radiologists graded the English-translated reports; 12 disagreements were resolved by a third. ChatGPT-4o was evaluated in standard mode, ChatGPT-5 in Thinking mode, and DeepSeek-R1 in Deep Thinking mode; model generation and inference mode therefore varied together. Exact-match accuracy with 95% Wilson confidence intervals (CIs), paired comparisons, and unweighted Gwet AC1 with 95% CIs were calculated. Management-relevant transitions and digital subtraction angiography (DSA) findings among high-risk downgrades were analyzed.

**Results:**

DeepSeek-R1 had an exact-match accuracy of 82.57% (95% CI, 80.89–84.13) and AC1 of 0.797 (95% CI, 0.778–0.816). ChatGPT-5 had an accuracy of 80.19% (95% CI, 78.43–81.84) and AC1 of 0.769 (95% CI, 0.749–0.789), whereas ChatGPT-4o had an accuracy of 53.62% (95% CI, 51.48–55.74) and AC1 of 0.460 (95% CI, 0.435–0.485). Cochran's *Q* test was significant (Q = 657.87; df = 2; *P* < 0.001); after Holm correction, DeepSeek-R1 also outperformed ChatGPT-5 in the paired comparison (*P* = 0.026), and both outperformed ChatGPT-4o (both *P* < 0.001). ChatGPT-4o downgraded 127/900 (14.11%) reference high-risk reports to CAD-RADS 3 or lower. Of these 127 cases, 65 underwent DSA within three months; DSA supported the original high-risk assessment in 56/65 (86.15%). The corresponding high-risk downgrade proportions were 37/900 (4.11%) for ChatGPT-5 and 23/900 (2.56%) for DeepSeek-R1.

**Conclusion:**

The DeepSeek-R1 Deep Thinking and ChatGPT-5 Thinking configurations achieved higher accuracy than ChatGPT-4o in standard mode. Because model generation and inference mode varied together, the results do not isolate a model-family effect. LLMs merit further evaluation for secondary structuring when no documented CAD-RADS category is available, but should not overwrite a radiologist-assigned category or independently determine management.

## 1 Introduction

Coronary computed tomography angiography (CCTA) is a noninvasive method for characterizing coronary stenosis and atherosclerotic burden (1, 2). CAD-RADS 2.0 standardizes CCTA communication and provides management considerations based on stenosis category, plaque burden, and modifiers (1). When a valid CAD-RADS category is documented by the reporting radiologist, it should be used directly. At our institution, however, explicit CAD-RADS categories were not routinely included in CCTA reports during the study period; reports primarily contained narrative descriptions of coronary plaque and stenosis severity. Automated secondary structuring of such narrative reports could support retrospective database construction, registry abstraction, cohort identification, quality assurance, and standardized communication when no explicit category is available.

Large language models (LLMs) have been applied in radiology to clinical information extraction (3–6), multilingual translation (7), report classification (8), and structured reporting and summarization (9). For the present use case, publicly accessible, non-fine-tuned interfaces are important objects of study because they require neither a locally labeled training set nor dedicated computational infrastructure or a model-deployment pipeline; they are immediately available and may already be used informally by clinicians. Evaluating these interfaces therefore characterizes performance at the point of access rather than the performance of an optimized institutional system (3, 10–12).

LLMs have also been evaluated across standardized radiology reporting systems, including BI-RADS, LI-RADS, Lung-RADS, and PI-RADS (13–16). A growing but methodologically heterogeneous literature has examined LLM-based CAD-RADS classification and structured extraction from CCTA reports (17–21). Collectively, these studies demonstrate feasibility but also show that results depend strongly on model generation, prompting strategy, report structure, case mix, input language, and the definition of the target output.

Important questions therefore remain regarding the performance of newer reasoning-enabled consumer interfaces under a common brief zero-shot, findings-only protocol; formal paired differences between models evaluated on the same reports; balanced category-specific performance across the full main-category spectrum; and the management-relevant consequences of classification errors. These questions are distinct from whether a fine-tuned or locally deployed model can be optimized for complete CAD-RADS 2.0 extraction.

Accordingly, we compared ChatGPT-4o, ChatGPT-5, and DeepSeek-R1 for assigning main CAD-RADS categories from findings-only CCTA text without an available structured CAD-RADS field. We evaluated exact-match accuracy, paired differences in correctness, agreement with an adjudicated radiologist reference standard, category-specific error patterns, and management-relevant upgrades and downgrades. An automatically derived category was considered a potential secondary structuring and communication aid when no documented category was available, not a replacement for the radiologist-authored report or a sole basis for management.

## 2 Methods

### 2.1 Study population

This retrospective diagnostic-accuracy study included CCTA reports generated at The Eighth Affiliated Hospital of Southern Medical University from March 2022 through October 2025. During this period, reports were screened in the institutional PACS. Only one CCTA examination per patient was included; the analytic dataset therefore comprised 2,100 reports from 2,100 unique patients. Reports were eligible when the findings section contained sufficient narrative information to assign a CAD-RADS category and were excluded when documented artifacts, coronary misregistration, or incomplete stenosis descriptions precluded category assignment. Eligible reports were provisionally stratified from the narrative stenosis descriptions and selected using nonrandom, category-specific quota sampling. The provisional strata were used only for sample construction and were not used as reference labels. After independent reader classification and adjudication, the final reference-standard dataset contained exactly 300 reports in each main category (CAD-RADS 0, 1, 2, 3, 4A, 4B, and 5), yielding 2,100 reports. The reports were exported from PACS for de-identification and analysis. Table 1 summarizes dataset construction and report characteristics, and Figure 1 shows the study workflow.

**Table 1:** Dataset construction and report characteristics.

Characteristic	Value
Study setting	Single-center retrospective diagnostic-accuracy study
Report period	March 2022–October 2025
Report source	Institutional PACS
Sampling design	Nonrandom quota-based category-balanced selection
Final analytic sample	2,100 CCTA reports from 2,100 unique patients
CAD-RADS composition	300 reports in each final adjudicated category: 0, 1, 2, 3, 4A, 4B, and 5
Source report language	Chinese
Human/model input language	English-translated findings-only text
Pre-existing structured CAD-RADS field	Not routinely documented; unavailable in the included findings-only inputs
Demographic and referral variables	Age, sex, and clinical indication were not retained in the findings-only analytic dataset

**Figure 1:**
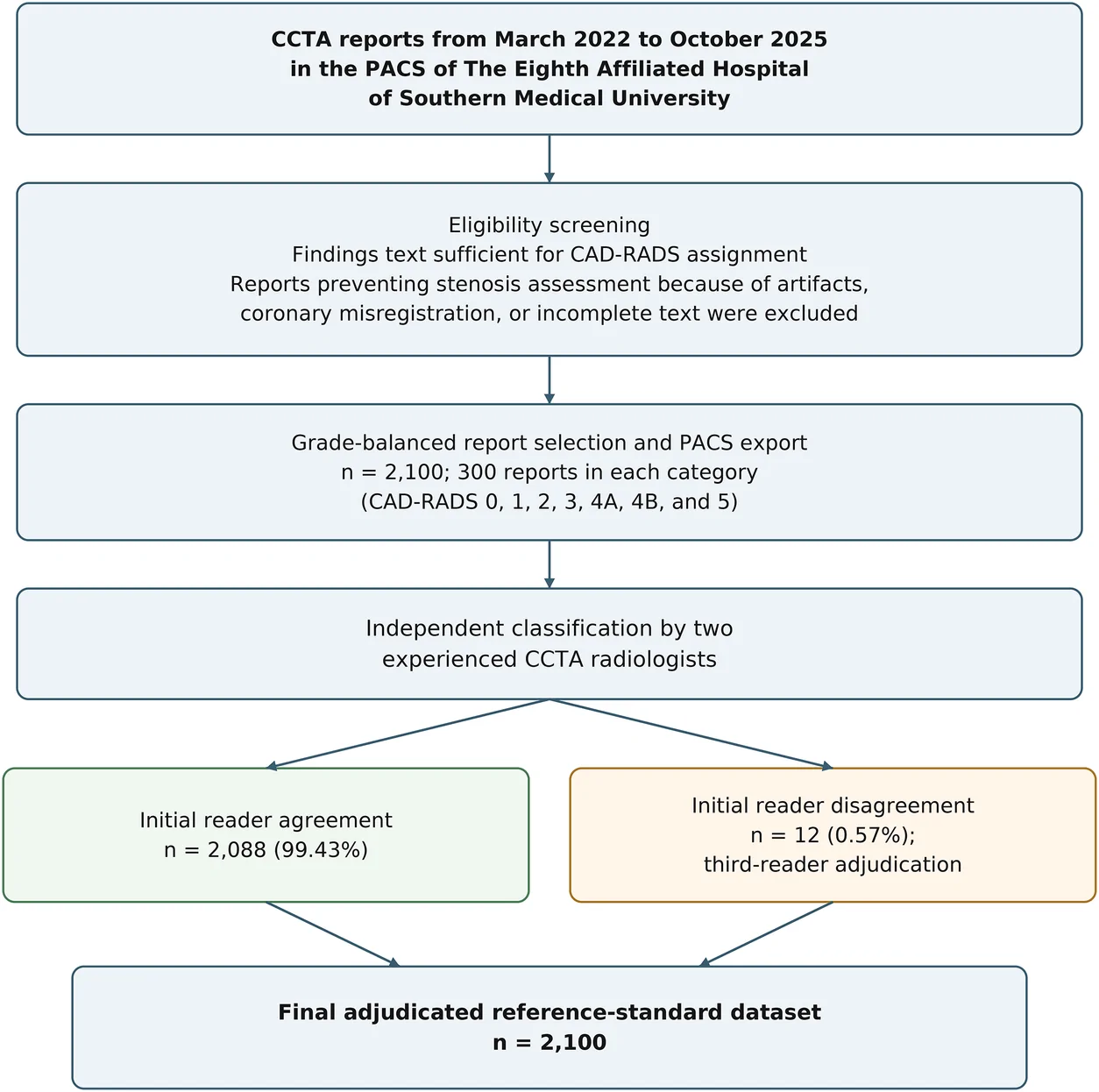
Study flow diagram showing PACS identification, eligibility screening, category-balanced selection, independent reader classification, and adjudication.

### 2.2 Study protocol

The original Chinese CCTA reports were exported to a structured spreadsheet. Patient identifiers, clinical history, and the diagnostic conclusion were removed before human classification and model inference. An explicit CAD-RADS category was not routinely recorded in the source reports, and PACS did not contain a reliable structured CAD-RADS field; consequently, no pre-existing category was available for systematic use as either the reference standard or a secondary comparator. Report-level categories were assigned according to CAD-RADS 2.0 logic: CAD-RADS 0, no plaque or stenosis; 1, 1%–24%; 2, 25%–49%; 3, 50%–69%; 4A, 70%–99% stenosis in one or two vessels; 4B, left-main stenosis of at least 50% or 70%–99% stenosis in all three major epicardial vessels; and 5, total occlusion. Assignment therefore incorporated maximum stenosis severity, left-main involvement, the number of severely stenosed vessels, and occlusion rather than the single most severe percentage alone (1).

The source reports were Chinese-language narrative CCTA reports authored during routine clinical care. After export and de-identification, all 2,100 findings sections were translated into English by a medical translator with 10 years of experience. The same fixed English translation was provided to both human readers and all three models. Two radiologists, each with 10 years of CCTA experience, independently reviewed the English-translated findings-only text without access to images, diagnostic conclusions, model outputs, or auxiliary tools. The 12 discordant reports were reviewed by a third radiologist with 10 years of CCTA experience, who assigned the final category. The reference standard was the concordant category when the first two readers agreed and the third reader's category when they disagreed. Reader agreement statistics are reported in Table 4; the third reader was not included in a whole-cohort pairwise analysis because only discordant cases were reviewed.

ChatGPT-4o and ChatGPT-5 (OpenAI), together with DeepSeek-R1 (DeepSeek), were evaluated without study-specific fine-tuning through their consumer web interfaces; no API was used. Detailed access dates, displayed model identifiers, inference modes, unavailable inference controls, and run conditions are summarized in Table 2. Before inference, the prompt and scoring rule were fixed and were not optimized on study cases. The English-translated findings section and the standardized prompt below were entered in a new independent chat for each report. Each report was submitted once to each model; no feedback, correction, few-shot examples, external retrieval, web search, browsing, auxiliary tools, or conversational history was used. The complete prompt was: ‘Assume you are a radiologist. The following is the description section of a patient's CCTA imaging report. Please classify it according to the CAD-RADS grading standard into CAD-RADS 0, 1, 2, 3, 4A, 4B, or 5.’ The prespecified output was a single main category; plaque burden, modifiers, and management recommendations were not requested or scored. When an output included explanatory text, scoring was based on the explicitly stated single category and the explanation was ignored. Because the configurations in Table 2 differed jointly in model generation and inference mode, these factors were not independently varied and their effects could not be separated.

**Table 2:** Model access and inference details.

Model	Interface/identifier	Evaluation dates	Inference controls	Runs/determinism
ChatGPT-4o	ChatGPT web interface; no API; displayed label: ChatGPT-4o; backend snapshot not disclosed	15 October–30 November 2025	Temperature, top-p, maximum output tokens, and seed not user-configurable/not disclosed; standard web-interface mode with no reasoning option selected	One run per report in a new independent chat; deterministic output not enforceable; repeatability not assessed
ChatGPT-5	ChatGPT web interface; no API; displayed label: ChatGPT-5; backend snapshot not disclosed	15 October–30 November 2025	Temperature, top-p, maximum output tokens, and seed not user-configurable/not disclosed; Thinking mode	One run per report in a new independent chat; deterministic output not enforceable; repeatability not assessed
DeepSeek-R1	DeepSeek web interface; no API; displayed label: DeepSeek-R1; backend snapshot not disclosed	15 October–30 November 2025	Temperature, top-p, maximum output tokens, and seed not user-configurable/not disclosed; Deep Thinking mode	One run per report in a new independent chat; deterministic output not enforceable; repeatability not assessed

Model names are the labels displayed by the web interfaces rather than immutable backend snapshot identifiers. Because the interfaces did not expose a seed and each report was submitted once, deterministic generation and intra-model repeatability were not assessed.

### 2.3 Statistical analysis

The primary model-performance measure was exact-match accuracy, defined as the proportion of all 2,100 reports for which the model-assigned category exactly matched the adjudicated reference category. Two-sided 95% CIs for model accuracy and pre-adjudication human-reader exact agreement were calculated using the Wilson score method without continuity correction (binom package). Because all three models classified the same reports, their paired binary correct/incorrect outcomes were compared using Cochran's *Q* test. Following a significant global test, pairwise two-sided exact McNemar tests were performed with Holm correction for three comparisons. Human interreader agreement before adjudication and model–reference agreement were quantified using unweighted Gwet AC1 with 95% CIs (irrCAC package), which is less sensitive than Cohen kappa to prevalence and marginal imbalance (22). An unweighted coefficient was used because the agreement endpoint required exact category identity; the ordinal and management-related severity of errors was evaluated separately in the transition analyses. Analyses were conducted in R version 4.6.0 (R Foundation for Statistical Computing, Vienna, Austria) (23).

For management-oriented error analysis, categories were grouped *a priori* as low (CAD-RADS 1–2), intermediate (CAD-RADS 3), and high (CAD-RADS 4A–5); CAD-RADS 0 was retained as a separate no-disease category. We distinguished any ordinal category change from a transition across these management-oriented groups. For reference high-risk cases downgraded by an LLM to CAD-RADS 3 or lower, the hospital record was reviewed for digital subtraction angiography (DSA) within three months after CCTA. A downgrade was considered unsupported by angiography when DSA demonstrated stenosis severity consistent with the adjudicated high-risk CCTA category. Because angiography was performed selectively, DSA findings were analyzed only within the verified subset and were not treated as verification of all downgraded cases.

## 3 Results

### 3.1 Overall accuracy and agreement

DeepSeek-R1 had the highest exact-match accuracy at 82.57% (1,734/2,100; 95% CI, 80.89–84.13), followed by ChatGPT-5 at 80.19% (1,684/2,100; 95% CI, 78.43–81.84) and ChatGPT-4o at 53.62% (1,126/2,100; 95% CI, 51.48–55.74) (Table 3). The paired global comparison was significant (Cochran's Q = 657.87; df = 2; *P* < 0.001). After Holm correction, exact McNemar tests showed that DeepSeek-R1 outperformed ChatGPT-5 (*P* = 0.026) and that both DeepSeek-R1 and ChatGPT-5 outperformed ChatGPT-4o (both *P* < 0.001). Human interreader and model–reference agreement statistics are summarized in Table 4.

**Table 3:** Classification accuracy of three LLMs across main CAD-RADS categories.

Model	Correctly classified cases	Accuracy	95% CI (Wilson)
ChatGPT-4o	1,126	53.62%	51.48–55.74
ChatGPT-5	1,684	80.19%	78.43–81.84
DeepSeek-R1	1,734	82.57%	80.89–84.13

Accuracy is the exact-match proportion among all 2,100 reports. The paired global comparison used Cochran's *Q* test (Q = 657.87; df = 2; *P* < 0.001). Pairwise two-sided exact McNemar tests with Holm correction showed *P* < 0.001 for ChatGPT-4o versus ChatGPT-5, *P* < 0.001 for ChatGPT-4o versus DeepSeek-R1, and *P* = 0.026 for ChatGPT-5 versus DeepSeek-R1. CI, confidence interval; CAD-RADS, coronary artery disease reporting and data system; LLM, large language model.

**Table 4:** Agreement between radiologists and three LLMs in main CAD-RADS category assignment.

Comparison	Unweighted AC1 (95% CI)
Reader 1 vs reader 2 (before adjudication)	0.987 (0.973–1.000)
Adjudicated reference vs ChatGPT-4o	0.460 (0.435–0.485)
Adjudicated reference vs ChatGPT-5	0.769 (0.749–0.789)
Adjudicated reference vs DeepSeek-R1	0.797 (0.778–0.816)

AC1, Gwet agreement coefficient; CI, confidence interval. The two initial readers agreed exactly in 2,088/2,100 reports (99.43%; Wilson 95% CI, 99.00–99.67); reader–reader AC1 was calculated before adjudication. Model comparisons use the final adjudicated radiologist reference standard.

### 3.2 Category-specific error patterns

ChatGPT-4o showed a tendency to overgrade lower categories. It correctly classified 18.0% of reference CAD-RADS 1 reports, while 77.7% were assigned CAD-RADS 2. For reference CAD-RADS 2 reports, 63.3% were exact matches and 35.0% were assigned CAD-RADS 3. At higher categories, category downgrades were more frequent than upgrades for CAD-RADS 3, 4A, and 4B (Figures 2,3).

**Figure 2:**
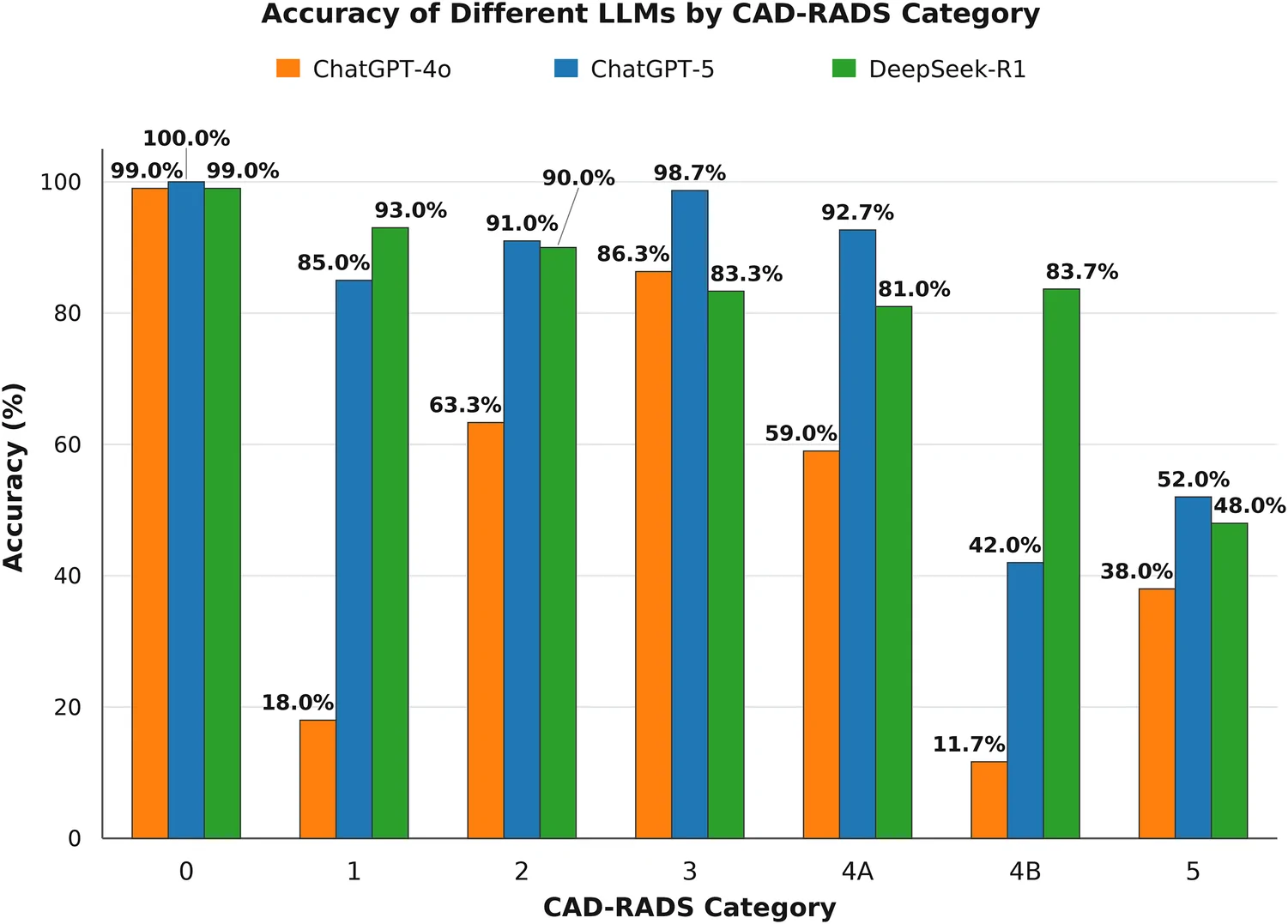
Classification accuracy of ChatGPT-4o, ChatGPT-5, and deepSeek-R1 for each CAD-RADS category.

**Figure 3:**
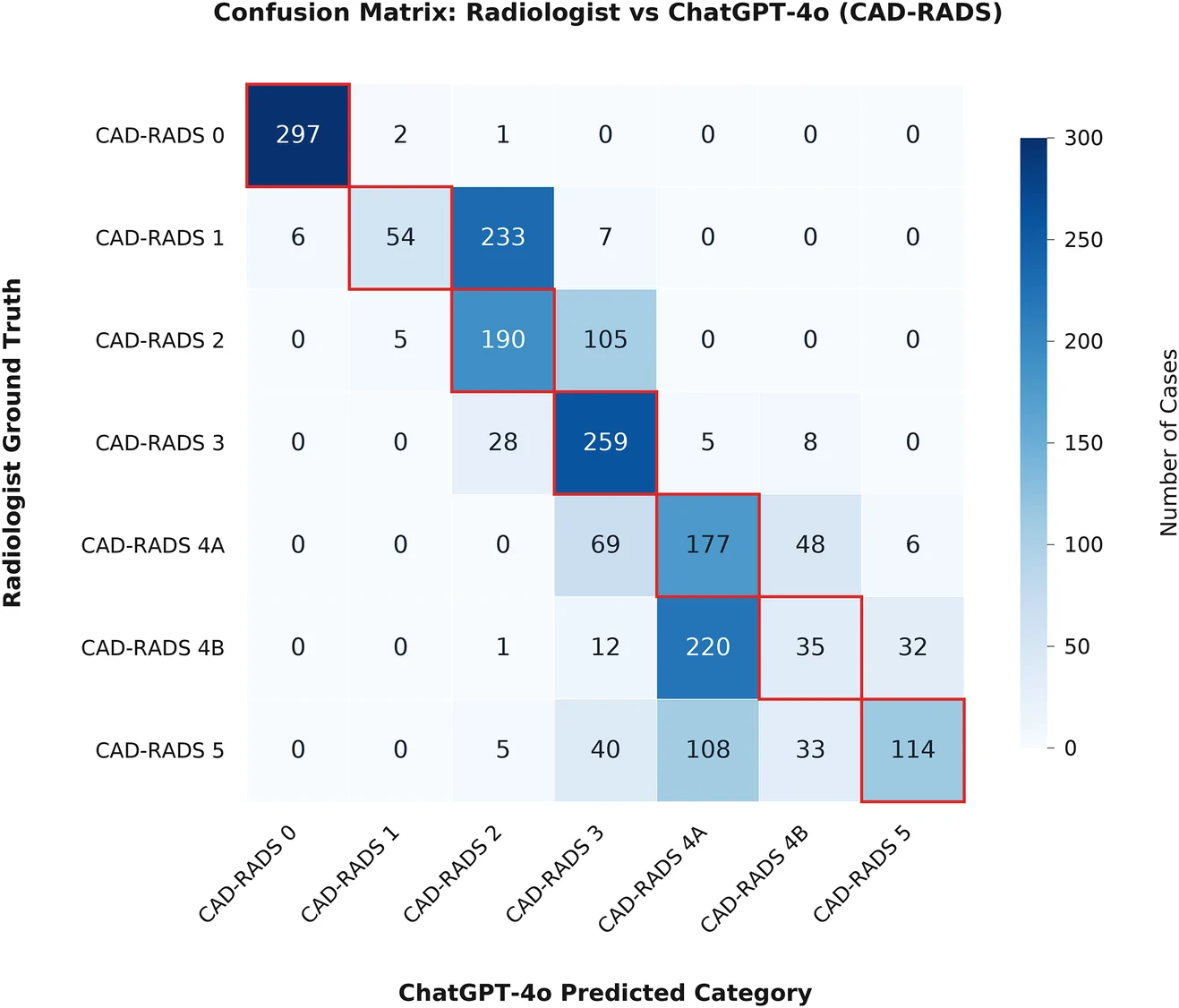
Agreement matrix for English-translated reports graded by ChatGPT-4o, showing overestimation at lower categories and underestimation at higher categories.

ChatGPT-5 had its highest category-specific performance for CAD-RADS 3 but lower exact-match accuracy for CAD-RADS 4B (42.0%) and CAD-RADS 5 (52.0%). The most common errors were assigning some 4B reports to 4A and assigning some occlusion reports to 4A or 4B (Figures 2,4).

**Figure 4:**
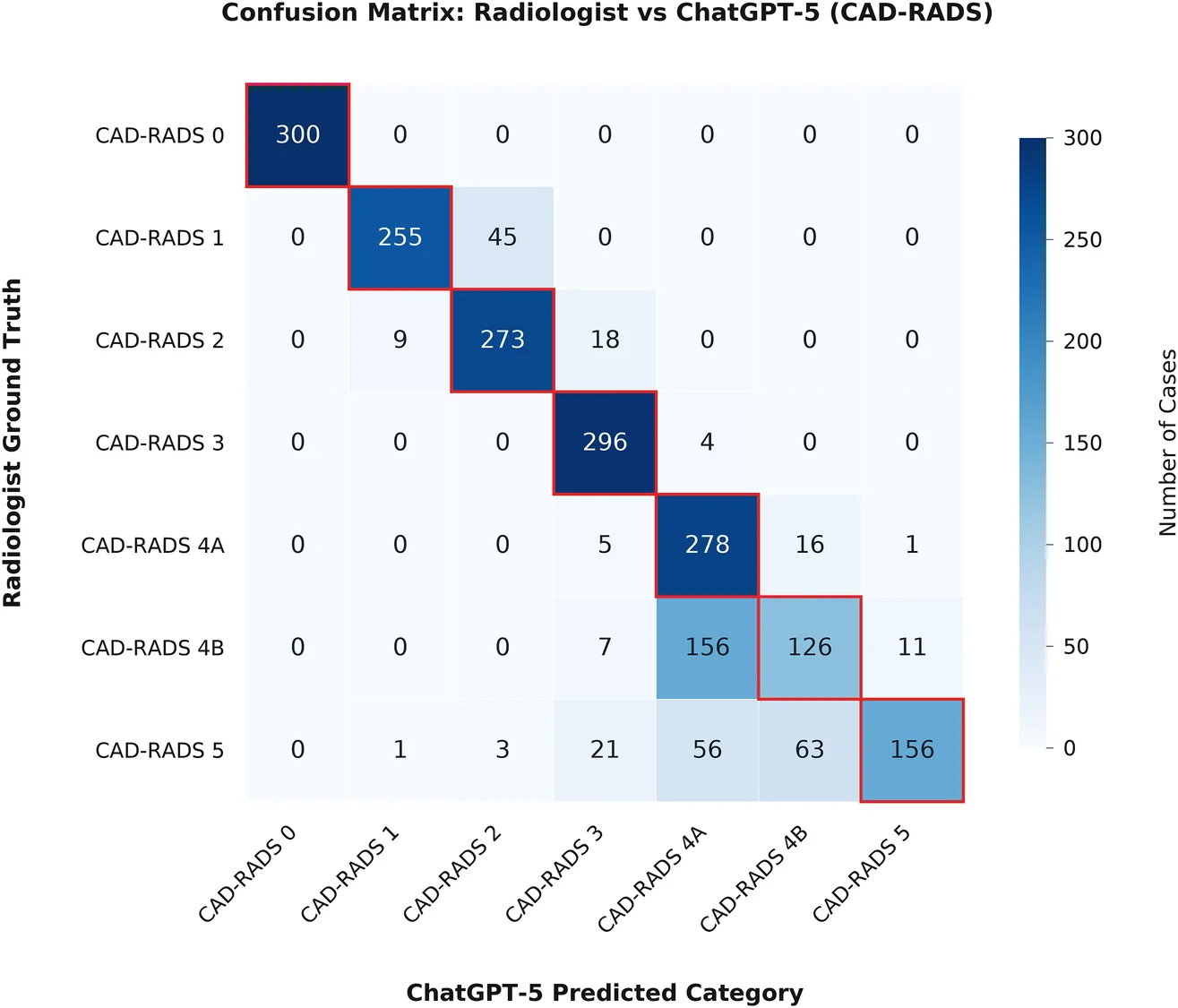
Agreement matrix for English-translated reports graded by ChatGPT-5.

DeepSeek-R1 had higher category-specific accuracy than ChatGPT-4o in most categories; CAD-RADS 3 was an exception, with 14.7% of reference category 3 reports assigned to 4A (Figures 2,5).

**Figure 5:**
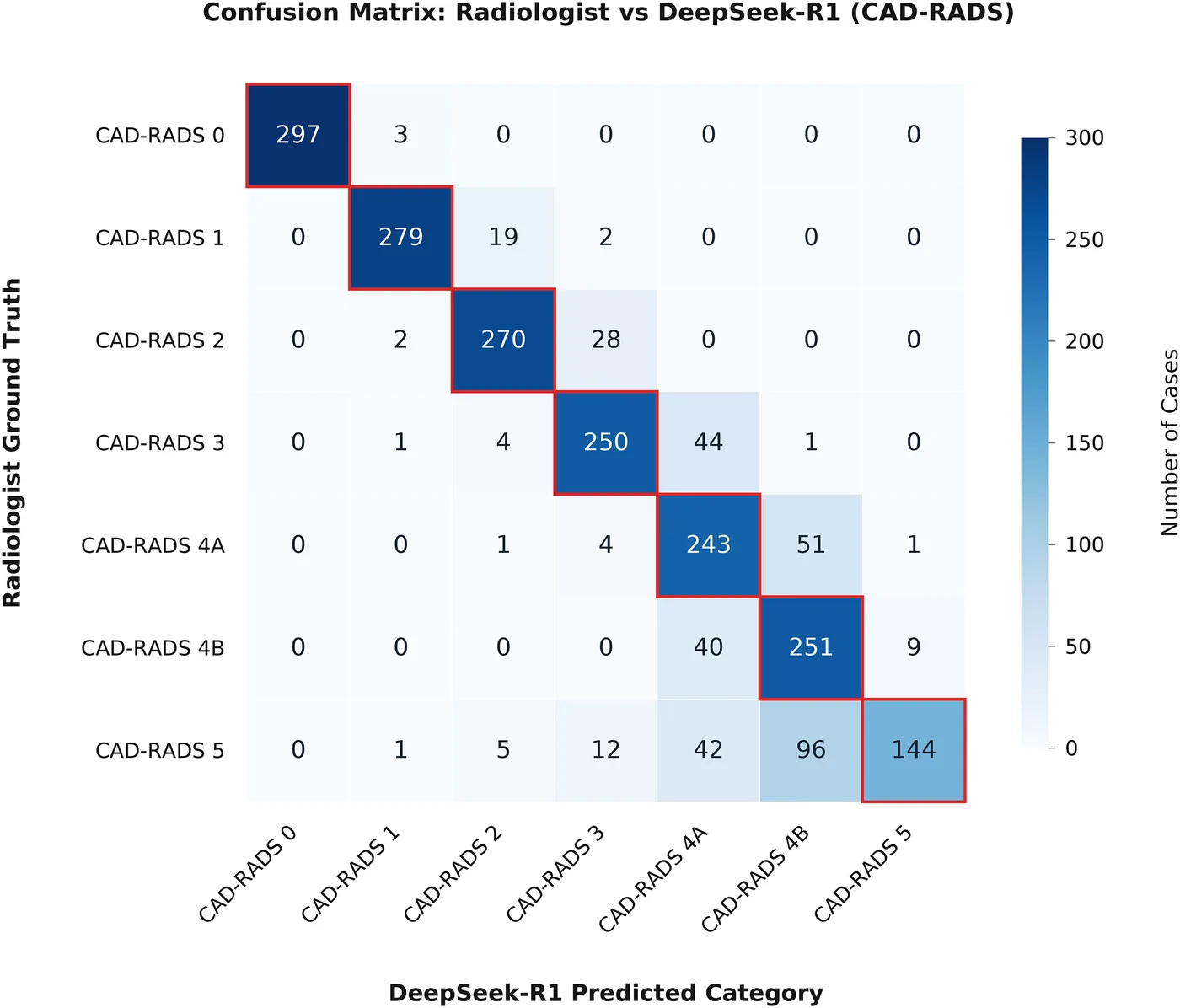
Agreement matrix for English-translated reports graded by deepSeek-R1.

### 3.3 Analysis by clinical management grouping

Management-relevant category transitions are summarized in Table 5 and Figures 6–8.

**Table 5:** Category transitions, management-group changes, and DSA verification of high-risk downgrades.

Transition	ChatGPT-4o	ChatGPT-5	DeepSeek-R1
Any category upgrade	447/2,100 (21.29%)	95/2,100 (4.52%)	158/2,100 (7.52%)
CAD-RADS 0 to any higher category	3/2,100 (0.14%)	0/2,100 (0.00%)	3/2,100 (0.14%)
CAD-RADS 1/2 to CAD-RADS 3	112/2,100 (5.33%)	18/2,100 (0.86%)	30/2,100 (1.43%)
CAD-RADS 3 to CAD-RADS 4A/4B/5	13/2,100 (0.62%)	4/2,100 (0.19%)	45/2,100 (2.14%)
CAD-RADS 1/2 to CAD-RADS 4A/4B/5	0/2,100 (0.00%)	0/2,100 (0.00%)	0/2,100 (0.00%)
Any category downgrade	527/2,100 (25.10%)	321/2,100 (15.29%)	208/2,100 (9.90%)
Any higher category to CAD-RADS 0	6/2,100 (0.29%)	0/2,100 (0.00%)	0/2,100 (0.00%)
CAD-RADS 3 to CAD-RADS 1/2	28/2,100 (1.33%)	0/2,100 (0.00%)	5/2,100 (0.24%)
CAD-RADS 4A/4B/5 to CAD-RADS 1/2	6/2,100 (0.29%)	4/2,100 (0.19%)	7/2,100 (0.33%)
CAD-RADS 4A/4B/5 to CAD-RADS 3	121/2,100 (5.76%)	33/2,100 (1.57%)	16/2,100 (0.76%)
Reference CAD-RADS 4A/4B/5 assigned CAD-RADS 0–3	127/2,100 (6.05%)	37/2,100 (1.76%)	23/2,100 (1.10%)
DSA-verified high-risk downgrades	65/127 (51.18%)	13/37 (35.14%)	7/23 (30.43%)
DSA did not support the downgrade	56/65 (86.15%)	11/13 (84.62%)	6/7 (85.71%)
DSA supported the downgrade	9/65 (13.85%)	2/13 (15.38%)	1/7 (14.29%)

All percentages in the category-transition rows use the full cohort (*n* = 2,100). The DSA-verification row uses all model-specific high-risk downgrades as denominators (127, 37, and 23), and the DSA-outcome rows use the verified subsets (65, 13, and 7). DSA findings should not be generalized to unverified downgrades. DSA, digital subtraction angiography.

**Figure 6:**
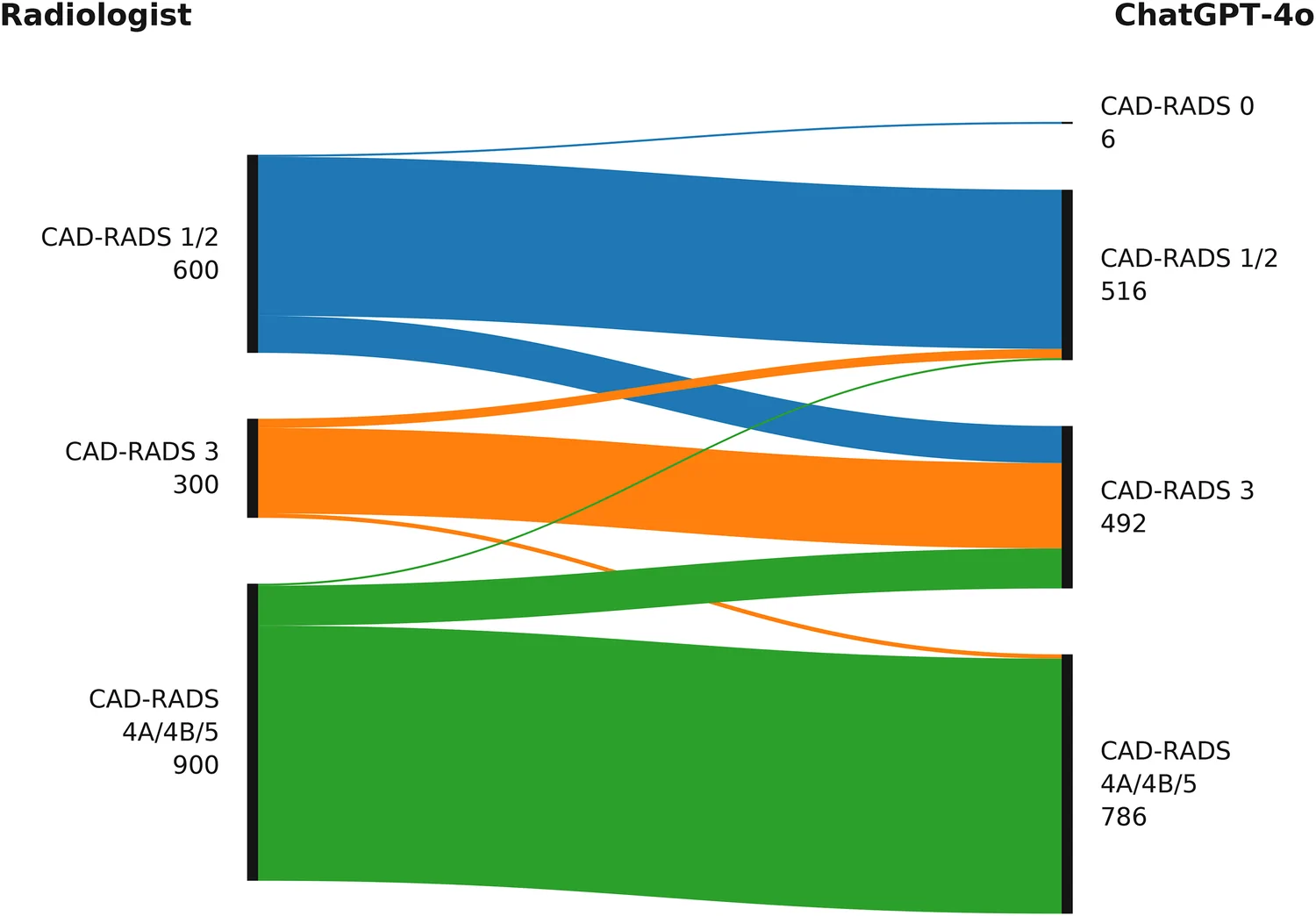
Sankey diagram showing CAD-RADS category changes between the adjudicated reference standard and ChatGPT-4o.

**Figure 7:**
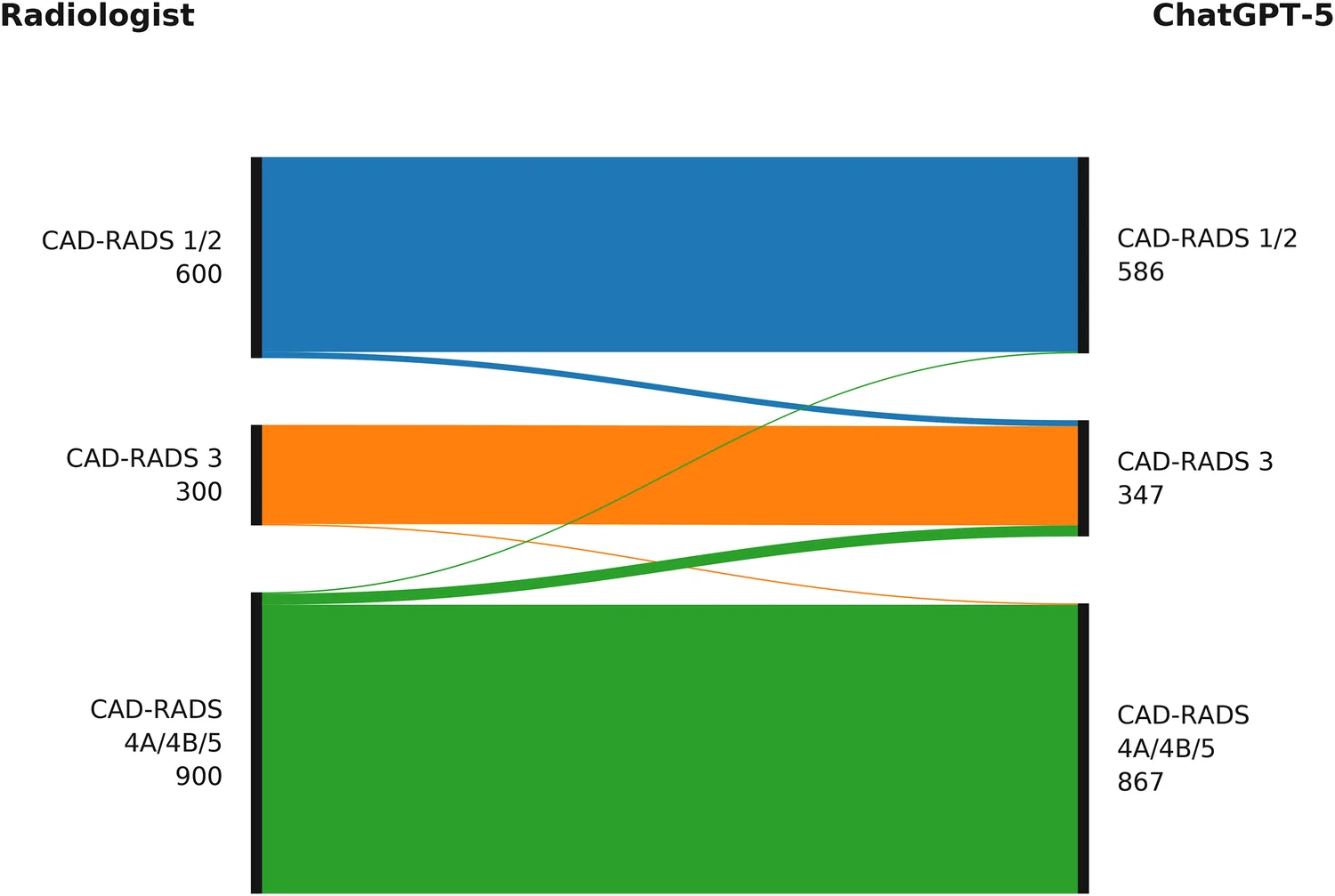
Sankey diagram showing CAD-RADS category changes between the adjudicated reference standard and ChatGPT-5.

**Figure 8:**
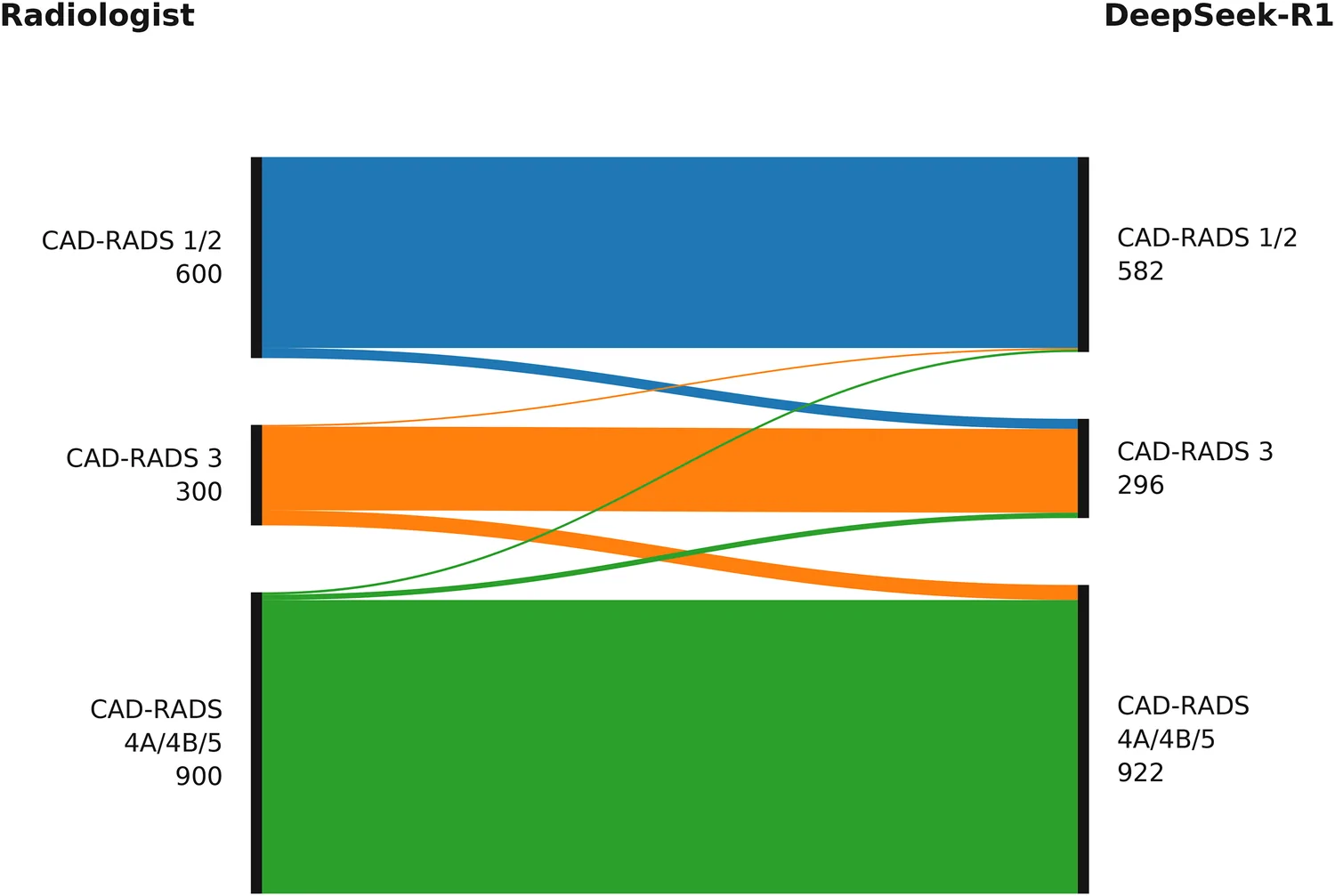
Sankey diagram showing CAD-RADS category changes between the adjudicated reference standard and deepSeek-R1.

Any ordinal category upgrade occurred in 447/2,100 (21.29%) ChatGPT-4o outputs, 95/2,100 (4.52%) ChatGPT-5 outputs, and 158/2,100 (7.52%) DeepSeek-R1 outputs. Low-to-intermediate transitions occurred in 112/2,100 (5.33%), 18/2,100 (0.86%), and 30/2,100 (1.43%), respectively. Intermediate-to-high transitions were most frequent for DeepSeek-R1 (45/2,100; 2.14%) (Table 5; Figures 6–8).

Any ordinal category downgrade occurred in 527/2,100 (25.10%) ChatGPT-4o outputs, 321/2,100 (15.29%) ChatGPT-5 outputs, and 208/2,100 (9.90%) DeepSeek-R1 outputs. Among the 900 reference high-risk reports, 127 (14.11%) were downgraded to CAD-RADS 3 or lower by ChatGPT-4o, 37 (4.11%) by ChatGPT-5, and 23 (2.56%) by DeepSeek-R1. DSA within three months was available for 65/127, 13/37, and 7/23 of these downgraded cases, respectively. Within these selected DSA subsets, angiography supported the adjudicated high-risk CCTA assessment in 56/65 (86.15%), 11/13 (84.62%), and 6/7 (85.71%), respectively. These subset proportions should not be generalized to all downgraded cases (Table 5).

## 4 Discussion

DeepSeek-R1 achieved the highest exact-match accuracy (82.57%; AC1, 0.797), followed by ChatGPT-5 (80.19%; AC1, 0.769) and ChatGPT-4o (53.62%; AC1, 0.460). The paired analysis confirmed differences between all three tested configurations, including the smaller difference between DeepSeek-R1 and ChatGPT-5 (Holm-adjusted *P* = 0.026). The dominant management-relevant error pattern was downgrading of reference high-risk reports, most pronounced for ChatGPT-4o (127/900, 14.11%). All model–reference AC1 values were below the pre-adjudication interreader AC1 of 0.987, indicating that even the two stronger configurations did not approach the agreement achieved by the experienced human readers.

### 4.1 Performance in context of prior CAD-RADS LLM studies

Prior studies evaluated related report-to-CAD-RADS tasks, but used different model generations, prompts, datasets, and output targets. Silbergleit et al. reported 87% accuracy for ChatGPT-4o (17), and Arnold et al. reported 93% accuracy for complete CAD-RADS 2.0 scoring with GPT-4o (18), both higher than the 53.62% observed here. Prompting strategy is the leading explanation for much of this discrepancy. Both studies provided more extensive in-context task guidance, whereas our deliberately brief zero-shot prompt neither reproduced the CAD-RADS decision rules nor supplied worked examples. Consistent with this interpretation, Min et al. demonstrated substantial prompt-dependent changes (19), and Kaya reported increases from 14% to 98% for ChatGPT o1 and from 8% to 93% for DeepSeek-R1 after few-shot prompting (20). These findings make prompt design a more direct explanation than an undifferentiated list of model and dataset differences, although the present study did not experimentally isolate the prompt effect.

Lorusso et al. reported the highest accuracy with DeepSeek V (91.83%), closely followed by GPT-4o (91.36%) (21). The present study extends this literature through a larger category-balanced cohort, formal paired comparisons, and analysis of management-relevant upgrades and downgrades with angiographic review in a clinically selected subset. The inclusion of 300 reports in each category also enabled stable category-specific assessment of less prevalent high-risk classes.

The paired results should nevertheless be interpreted as comparisons among the tested model–mode configurations. ChatGPT-4o was evaluated in standard mode, whereas ChatGPT-5 and DeepSeek-R1 were evaluated in reasoning-enabled modes; model generation and inference mode therefore varied together. Consequently, the observed differences cannot be attributed to model family, provider, or reasoning mode in isolation and should not be interpreted as an intrinsic ranking of vendors. DeepSeek-R1 is an open-weight, reasoning-oriented model, but the present study evaluated its consumer web implementation rather than a locally fixed checkpoint (24).

### 4.2 Clinical risk grouping: implications of upgrading and downgrading errors

ChatGPT-4o produced the largest proportion of management-relevant high-risk downgrades. Such errors could delay specialist assessment if an automatically derived category were used without reference to the original radiologist-authored report. Among the selected downgraded cases that underwent DSA within three months, angiography supported the adjudicated high-risk CCTA category in 56/65 (86.15%) for ChatGPT-4o, 11/13 (84.62%) for ChatGPT-5, and 6/7 (85.71%) for DeepSeek-R1.

Upgrades and downgrades are not clinically equivalent. Overgrading may lead to unnecessary specialist referral, functional testing, or invasive evaluation, whereas undergrading may delay appropriate assessment of clinically important disease. Reporting the direction and magnitude of category transitions is therefore more informative for implementation than overall accuracy alone.

### 4.3 Observed rule-sensitive error patterns

The concentration of errors around CAD-RADS 4B and 5 suggests difficulty integrating multiple rule elements—including left-main prioritization, the three-vessel criterion, and the distinction between severe stenosis and total occlusion—rather than simply extracting the highest reported stenosis percentage. ChatGPT-4o's frequent shifts from category 1 to 2 and from category 2 to 3 suggest imprecise threshold mapping at lower and intermediate boundaries, whereas DeepSeek-R1's category 3-to-4A upgrades indicate a different boundary-related pattern. These findings support separating flexible vessel-level information extraction from deterministic application of the final CAD-RADS rules.

### 4.4 Clinical relevance and potential workflow integration

The clinical value of automated CAD-RADS classification should be considered primarily at the scale of secondary report structuring rather than the time required to grade an individual report. Experienced cardiovascular radiologists can assign a CAD-RADS category rapidly during routine interpretation, and requiring case-by-case verification of every automatically generated category could negate the efficiency of automation. Oversight should therefore be proportionate to the intended use rather than assumed to be identical across all applications.

A practical non–patient-facing application would be the large-scale secondary conversion of narrative reports lacking an explicit category for retrospective database construction, registry abstraction, cohort identification, quality assurance, and standardization of legacy or externally generated reports. After validation in the target dataset, such applications could be governed by periodic sampling and error monitoring rather than universal specialist review.

Use within a clinical information system would require prospective validation and stronger safeguards. Any automatically derived category should be clearly identified as such and displayed only as an adjunct to the original radiologist-authored report, which remains the authoritative source. It should not serve as the sole basis for management. Outputs that could influence management, conflict with the narrative findings, or contain high-risk or ambiguous features should undergo targeted specialist review. Future studies should evaluate whether risk-adapted oversight improves report comprehension, extraction efficiency, and appropriate referral without increasing automation bias.

### 4.5 Future directions for optimization

Near-term optimization should prioritize transparent fixed prompts that include the CAD-RADS 2.0 rules, representative few-shot examples, constrained output formats, and retrieval of the current guideline. CAD-RADS-specific studies have shown large gains from few-shot and chain-of-thought prompting (19, 20), and retrieval-augmented approaches can further improve extraction of categories, plaque burden, modifiers, and management recommendations (25). A prospective prompt-ablation study should apply brief zero-shot, rule-augmented, and few-shot prompts to the same reports under the same immutable model snapshot and inference mode; this matched design is necessary to isolate prompt effects from model updates and reasoning-mode differences.

A hybrid architecture may be particularly suitable for future clinical evaluation, with an LLM used to extract vessel-level findings from heterogeneous narrative text and a deterministic rule engine used to assign the final CAD-RADS category. Rule-based safety triggers could flag left-main stenosis, multivessel severe disease, total occlusion, or internal contradictions for targeted review (1). This approach could preserve flexible language extraction while reducing threshold and multivessel-rule errors. Future studies should also prospectively evaluate the complete CAD-RADS 2.0 framework using a modifier-inclusive prompt, complete reference annotations for plaque burden and all modifiers, and explicit rules for omitted and multiple outputs. Longer-term work may combine text with imaging and clinical context, but such systems require prospective validation, calibration, privacy safeguards, and proportionate human oversight.

### 4.6 Limitations

This study has several limitations. First, it was a single-center retrospective study of English translations of Chinese findings sections without images or clinical context; the results therefore assess text classification rather than primary CCTA interpretation. Although the same fixed English translation was provided to both human readers and all models, translation may create model-specific bias because multilingual training and language-dependent performance differ among model families. English input could favor the ChatGPT configurations, remove a potential Chinese-language advantage for DeepSeek-R1, or influence the models in other directions; the observed ranking is therefore conditional on the translated English input. Age, sex, and referral indications were not retained in the findings-only analytic dataset. Second, the nonrandom quota-based equal-frequency design overrepresents high categories relative to routine practice and may introduce selection bias; overall accuracy and management-transition percentages are not prevalence-weighted estimates of clinical performance. Third, angiographic verification was available only for clinically selected subsets of high-risk downgrades, so the DSA corroboration proportions cannot be generalized to unverified cases.

Fourth, each report was submitted once, and intra-model repeatability was not assessed. Fifth, the consumer interfaces did not expose immutable backend snapshots, numerical decoding parameters, or random seeds and may have been updated over time, limiting reproducibility. Sixth, model generation and inference mode varied together: ChatGPT-4o was evaluated in standard mode, whereas ChatGPT-5 and DeepSeek-R1 were evaluated in reasoning modes. Seventh, prompting strategy was not experimentally varied; although prior evidence makes the brief zero-shot prompt the leading explanation for the lower ChatGPT-4o accuracy relative to in-context studies, a causal prompt effect cannot be established without a matched prompt-ablation experiment under the same model snapshot. Eighth, only the main CAD-RADS category was evaluated; plaque burden and modifiers were neither systematically requested nor prospectively annotated, and occasional unsolicited modifier outputs could not support unbiased performance estimates. Ninth, both initial human readers had the same level of CCTA experience, precluding experience-stratified interobserver analysis. Tenth, we did not evaluate clinician comprehension, data-extraction efficiency, referral decisions, or risk-adapted oversight strategies; the proposed workflows require prospective validation.

## 5 Conclusion

In a category-balanced, findings-only task, the DeepSeek-R1 Deep Thinking and ChatGPT-5 Thinking configurations achieved higher exact-match accuracy and model–reference AC1 than ChatGPT-4o in standard mode, and the paired difference between DeepSeek-R1 and ChatGPT-5 was also statistically significant. Nevertheless, all model–reference AC1 values remained below the human interreader AC1, and all models produced clinically relevant errors. Because model generation, inference mode, and input language were not independently varied, the results apply to the tested English-language configurations rather than establishing an intrinsic model-family ranking. These findings support further evaluation of LLMs for large-scale secondary structuring of narrative reports when an explicit CAD-RADS category is unavailable, using quality-control requirements matched to the intended use. They do not support replacing a documented radiologist-assigned category or basing patient management on unverified model output.

## Data Availability

The raw data supporting the conclusions of this article will be made available by the authors, without undue reservation.
